# Ferroptosis in Acute Central Nervous System Injuries: The Future Direction?

**DOI:** 10.3389/fcell.2020.00594

**Published:** 2020-07-15

**Authors:** Lesang Shen, Danfeng Lin, Xiaoyi Li, Haijian Wu, Cameron Lenahan, Yuanbo Pan, Weilin Xu, Yiding Chen, Anwen Shao, Jianmin Zhang

**Affiliations:** ^1^Department of Breast Surgery, The Second Affiliated Hospital, Zhejiang University School of Medicine, Hangzhou, China; ^2^Department of Surgical Oncology, The Second Affiliated Hospital, Zhejiang University School of Medicine, Hangzhou, China; ^3^Department of Nuclear Medicine and PET-CT Center, The Second Hospital, Zhejiang University School of Medicine, Hangzhou, China; ^4^Department of Neurosurgery, The Second Affiliated Hospital, Zhejiang University School of Medicine, Hangzhou, China; ^5^Burrell College of Osteopathic Medicine, Las Cruces, NM, United States; ^6^Center for Neuroscience Research, School of Medicine, Loma Linda University, Loma Linda, CA, United States

**Keywords:** ferroptosis, iron metabolism, lipid metabolism, stroke, traumatic brain injury, spinal cord injury, therapy

## Abstract

Acute central nervous system (CNS) injuries, such as stroke, traumatic brain injury (TBI), and spinal cord injury (SCI) present a grave health care challenge worldwide due to high morbidity and mortality, as well as limited clinical therapeutic strategies. Established literature has shown that oxidative stress (OS), inflammation, excitotoxicity, and apoptosis play important roles in the pathophysiological processes of acute CNS injuries. Recently, there have been many studies on the topic of ferroptosis, a form of regulated cell death characterized by the accumulation of iron-dependent lipid peroxidation. Some studies have revealed an emerging connection between acute CNS injuries and ferroptosis. Ferroptosis, induced by the abnormal metabolism of lipids, glutathione (GSH), and iron, can accelerate acute CNS injuries. However, pharmaceutical agents, such as iron chelators, ferrostatin-1 (Fer-1), and liproxstatin-1 (Lip-1), can inhibit ferroptosis and may have neuroprotective effects after acute CNS injuries. However, the specific mechanisms underlying this connection has not yet been clearly elucidated. In this paper, we discuss the general mechanisms of ferroptosis and its role in stroke, TBI, and SCI. We also summarize ferroptosis-related drugs and highlight the potential therapeutic strategies in treating various acute CNS injuries. Additionally, this paper suggests a testable hypothesis that ferroptosis may be a novel direction for further research of acute CNS injuries by providing corresponding evidence.

## Introduction

Acute CNS injuries, including stroke, TBI, and SCI, are a major burden of morbidity and mortality worldwide (GBD, 2016, 2019). Each year, approximately 80 million individuals in the United States suffer a stroke. Moreover, deaths caused by stroke contribute to nearly 5% of all deaths in the United States. Ischemic stroke accounts for 87% of all strokes, with ICH comprising the remaining 10% (Benjamin et al., 2019). Another neurological disease worth mentioning is TBI, which has a global incidence of more than 50 million cases annually (Maas et al., 2017; Jiang et al., 2019). Regarding the mechanisms associated with acute CNS injuries, previous literature has shown that various mechanisms, including OS, inflammation, excitotoxicity, and apoptosis, play important roles in the pathophysiological processes of acute CNS, and targeting these mechanisms may provide neuroprotection (Roth et al., 2014; Chamorro et al., 2016; Duan et al., 2019; Nazemi et al., 2020). However, there is a lack of effective therapeutic strategies in treating long-term CNS injuries. Patients who survive CNS injuries often have long-term disabilities due to substantial neurological deficits and impaired tissue function, therefore requiring subsequent lifelong care. New therapeutic approaches are urgently required to improve outcomes of patients with acute CNS injuries. In recent years, there has been increasing interest in ferroptosis, suggesting a potential role of ferroptosis in acute CNS injuries and offering opportunities for novel pharmacological interventions, as ferroptosis can be modulated by small molecules (Friedmann Angeli et al., 2014; Lewerenz et al., 2018; Alim et al., 2019).

Ferroptosis, first observed in response to treatment of tumor cells via small-molecule chemical probes, is a newly identified form of regulated cell death characterized by the accumulation of iron-mediated lipid peroxides (Dixon et al., 2012). It differs from other programmed cell deaths (e.g., apoptosis, necrosis, and autophagy) at the morphological, biological, and genetic levels (Dixon et al., 2012). Regarding the function of ferroptosis within the tumor, it is associated with malignant transformation, cancer progression, and drug resistance [for review see Su et al. (2020)]. Moreover, ferroptosis regulation may be useful for anti-cancer therapy (Guo et al., 2019; Su et al., 2020). Although ferroptosis was first defined in cancer cells and has potential in cancer treatment, the latest experimental results have identified its role in the pathophysiology of acute organ injuries, such as acute kidney, lung, and brain injuries (Dixon et al., 2012; Friedmann Angeli et al., 2014; Kenny et al., 2019; Hu et al., 2020; Li Y.C. et al., 2020). More importantly, ferroptosis can cause neuronal cell death and neurological deficits in CNS injuries and human neurodegenerative diseases (Dixon et al., 2012; Morris et al., 2018). Therefore, targeting ferroptosis through effective anti-ferroptotic agents may provide direction for treating acute CNS injuries (Tuo et al., 2017; Zille et al., 2017; Kenny et al., 2019). So, what is the underlying mechanism of ferroptosis, and how does it affect acute CNS injuries?

## Discovery and Mechanisms of Ferroptosis

**FIGURE 1:**
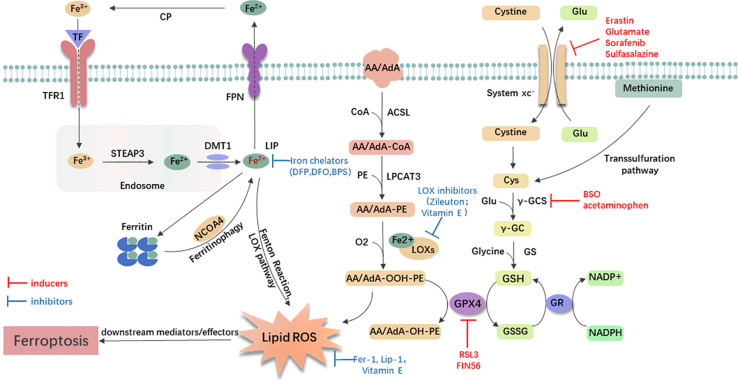
General mechanism of ferroptosis associated with lipid, amino acid, and iron metabolism. The inactive Fe^3+^ is delivered into the cell by TFR1 and reduced to Fe^2+^ in the endosome. Then, DMT1 transports Fe^2+^ to the labile iron pool (LIP). Autophagic degradation of ferritin (ferritinophagy) releases Fe^2+^ from ferritin, which is mediated by NCOA4. Fe^2+^ produces lipid ROS via the Fenton reaction and through the LOX pathway. Moreover, ACSL4 is required to activate polyunsaturated fatty acid, especially AA and AdA, to AA/AdA-CoA, then LPCAT3 catalyzes these derivatives and membrane PEs to form AA/AdA-PE, which are further converted into pro-ferroptotic lipid peroxidation under the activity of iron-containing LOXs. In conclusion, the Fenton reaction and oxidation of lipids facilitate the generation of lipid ROS, thus leading to ferroptosis. The system xc- is a cystine/glutamate antiporter. Intracellular cystine is reduced to cysteine for the biosynthesis of GSH. GPX4 converts two GSH molecules to GSSG each catalytic cycle to reduce lipid hydroperoxides, and then GSSG can be recycled back via GSH reductase in an NADPH-dependent manner. Ferroptosis inducers inhibit the GPX4-GSH-cysteine axis, thus inhibiting the reduction of lipid ROS. AA/AdA, arachidonic acid or adrenic acid; AA/AdA-CoA, arachidonic acid or adrenic acid coenzyme A; AA/AdA-PE, arachidonic acid or adrenic acid-phosphatidylethanolamine; AA/AdA-OOH-PE, arachidonic acid or adrenic acid-hydroperoxides-phosphatidylethanolamine; AA/AdA-OH-PE, arachidonic acid or adrenic acid-hydroxides-phosphatidylethanolamine; ACSL4, acyl-CoA synthetase long-chain family member 4; BSO, buthionine sulfoximine; CoA, coenzyme A; Cp, ceruloplasmin; Cys, L-cysteine; DMT1, divalent metal transporter 1; Fer-1, ferrostatin-1; FPN, ferroportin; γ-GC, gamma-glutamylcysteine; γ-GCS, gamma-glutamylcysteine synthetase; Glu, L-glutamate; GPX4, glutathione peroxidase 4; GR, glutathione reductase; GS, glutathione synthetase; GSH, reduced glutathione; GSSG, di-glutathione; LIP, labile iron pool; Lip-1, liproxstatin-1; LOX, lipoxygenase; LPCAT3, lysophosphatidylcholine acyltransferase 3; NCOA4, nuclear receptor coactivator 4; PE, phosphatidylethanolamine; RSL3, RAS-selective lethal 3; STEAP3, 6-transmembrane epithelial antigen of the prostate 3; TF, transferrin; TFR1, transferrin receptor 1.

Small-molecule probes are valuable tools for studying different types of regulated cell death (Gangadhar and Stockwell, 2007). During the identification of ferroptosis, there were two important chemical probes. Ferroptosis inducers, erastin, and RSL3, were discovered in a phenotypic small molecule-screening study (Dolma et al., 2003; Yang and Stockwell, 2008). Erastin, a synthetic compound, was capable of inducing non-apoptotic cell death to selectively kill HRAS-mutant engineered cancer cells, and in this process, there was no evidence of caspase activation or apoptotic hallmarks (Dolma et al., 2003; Yagoda et al., 2007). Another compound was RSL3, found in 2008, and capable of triggering a similar form of non-apoptotic and iron-dependent oxidative cell death (Yang and Stockwell, 2008). This erastin- and RSL3-induced cell death did not exhibit the morphological or biochemical features of apoptosis, and inhibition of necroptosis or autophagy had no effect on this mode of cell death (Wolpaw et al., 2011; Dixon et al., 2012; Yang et al., 2014). However, this manner of cell death could be prevented by the iron chelator, DFO, and antioxidants (e.g., Vitamin E) (Dolma et al., 2003; Yagoda et al., 2007; Yang and Stockwell, 2008). Therefore, the term “ferroptosis” was first proposed in 2012 to describe this novel iron-dependent non-apoptotic cell death (Dixon et al., 2012). Hallmark contributions of ferroptosis were well-displayed by Hirschhorn and Stockwell (2019) and Li J. et al. (2020) [for review see Hirschhorn and Stockwell (2019); Li J. et al. (2020)]. Hadian and Stockwell (2020) drew a SnapShot to provide an overview of ferroptosis-related pathways. Although the exact mechanisms of ferroptosis are still being explored, the initiation and execution of ferroptosis involve several biological processes, including lipid, GSH, and iron metabolism, as well as other regulatory processes (Dixon et al., 2012; Figure 1).

### Lipid Metabolism Related to Ferroptosis

Lipid metabolism is closely linked to the regulation of ferroptosis. The accumulation of lipid peroxidation seems to be a key process in the execution phase, in which PUFAs play an important role (Stockwell et al., 2017; Wenzel et al., 2017; Yamada et al., 2020). Usually, free PUFAs, especially AA and AdA are esterified to membrane phospholipids [mainly PUFA-containing phosphatidylethanolamines (PEs)]. With the presence of two lipid-metabolic enzymes, ACSL4 and LPCAT3, these membrane phospholipids undergo oxidation to drive ferroptosis (Doll et al., 2017; Kagan et al., 2017). The knockout of ACSL4 or loss of LPCAT3 resulted in significant resistance of certain non-neuronal cells to ferroptosis (Dixon et al., 2015; Yuan et al., 2016; Doll et al., 2017; Kagan et al., 2017). Following the generation of AA/AdA-PE, activated LOXs catalyze AA/AdA-PE into pro-ferroptotic lipid peroxidation AA/AdA-OOH-PE (Yang et al., 2016; Lei et al., 2019). The role of the LOXs in ferroptosis is also supported by a study indicating that genetic depletion or inhibition of LOXs by inhibitors [e.g., zileuton (Liu et al., 2015) and Vitamin E hydroquinone (Hinman et al., 2018)] could protect against ferroptosis in some cell types (Seiler et al., 2008; Yang et al., 2016). Recently, PEBP1 was shown to bind 15-LOX and alter the substrate specificity, changing it from free fatty acid to AA-PE, thereby promoting lipid oxidation (Wenzel et al., 2017). Furthermore, lipid peroxidation is thought to play a role in the final phase of ferroptosis, although the downstream mechanisms remain unclear (Lei et al., 2019). In one hypothesis, lipid peroxides may decompose into reactive toxic aldehydes, such as MDA or 4-HNEs. These decomposed substances react with proteins, nucleic acids, and membrane lipids to initiate ferroptosis (Domingues et al., 2013; Zhong and Yin, 2015). Dixon et al. also favored the hypothesis that showed that increased expression of AKRF1C genes could suppress ferroptosis by encoding aldoketoreductases to detoxify the end-products of lipid peroxidation (Dixon and Stockwell, 2014; Stockwell et al., 2017). As for inhibitors of lipid peroxidation, ferrostatins are the novel synthetic antioxidants that specifically trap lipid radicals and exert anti-ferroptotic function. Fer-1, the first-generation ferrostatin, prevents ferroptosis induced by erastin and RSL3 in HT1080 cells (Dixon et al., 2012). Lip-1 is another recently discovered ferroptosis inhibitor. It can prevent the accumulation of lipid ROS and inhibit erastin- or RSL3- induced ferroptosis *in vitro* (Friedmann Angeli et al., 2014). In conclusion, AA/AdA-related lipid metabolism can induce ferroptosis, and inhibiting LOXs or lipid peroxidation may have protective effects.

### Glutathione Metabolism Related to Ferroptosis

Previous studies have identified that two major mechanisms, the Se-dependent GPX4-GSH-cysteine axis (Friedmann Angeli et al., 2014; Yang et al., 2014; Friedmann Angeli and Conrad, 2018; Ingold et al., 2018) and the FSP1-ubiquinone (CoQ10)-NAD(P)H pathway (Bersuker et al., 2019; Doll et al., 2019), were associated with lipid peroxidation and ferroptotic cell death. Additionally, the FSP1-CoQ10-NAD(P)H pathway is a complementary system to the GPX4-GSH-cysteine axis for controlling ferroptosis. In this axis, key steps include cystine uptake via system xc-, reduction of cystine to cysteine, GSH biosynthesis, and GPX4-mediated reduction of phospholipid hydroperoxides to lipid alcohols. During the process, the cystine/glutamate antiporter (system xc-) which consists of the light-chain subunit xCT (SLC7A11) and the heavy-chain subunit CD98 (SLC3A2) exchanges intracellular glutamate for extracellular cystine at a ratio of 1:1. Cystine is then reduced to cysteine for GSH synthesis [for review see Xie et al. (2016)]. In this regard, several agents [e.g., glutamate and erastin (Dixon et al., 2012), sulfasalazine (Gout et al., 2001), and sorafenib (Dixon et al., 2014)] can inhibit the system xc- to cause the decreased acquisition of precursors and GSH depletion, ultimately leading to ferroptosis. Other agents, including BSO (Sun et al., 2018) and acetaminophen (Lorincz et al., 2015), were observed directly blocking GSH synthesis. Conversely, ferroptosis induced by cystine deprivation can be reversed by reagents that increase the level of intracellular cysteine/cystine. For example, an *in vitro* study showed that when in the presence of β-mercaptoethanol, the cells were able to constantly utilize cystine through a mixed disulfide of β-mercaptoethanol and cysteine (Ishii et al., 1981). In addition, the loss of cysteinyl-tRNA synthetase, as Hayano et al. (2016) indicated, could trigger the transsulfuration pathway and lead to inhibition of ferroptosis induced by cystine deprivation.

Glutathione peroxidases 4 is a type of selenoprotein that contains one selenocysteine at the active site and seven cysteines. It plays an important role in regulating ferroptosis, and its inhibition promotes ferroptosis (Yang et al., 2016). Regarded as the only GPX that can eliminate biomembrane lipid peroxidation, GPX4 has a unique ability in ferroptosis. It is capable of reducing the toxic, membranous lipid hydroperoxides into non-toxic lipid alcohols (Brigelius-Flohé and Maiorino, 2013; Yang et al., 2014). Increasing GPX4 has been shown to be beneficial in many models of disease by inhibiting ferroptosis (Lan et al., 2020; Shen et al., 2020). However, knockdown or inactivation of GPX4 contributes to the accumulation of lipid peroxidation and initiation of ferroptosis (Park et al., 2019; Ye et al., 2020). For example, RSL3 directly inactivated GPX4 by covalently binding to selenocysteine to trigger ferroptosis (Yang et al., 2014, 2016), and FIN56 promoted degradation of GPX4 (Shimada et al., 2016).

### Iron Metabolism Related to Ferroptosis

Besides lipid and GSH metabolism, the essential trace element for life, iron, is indispensable for the execution of ferroptosis (Dixon and Stockwell, 2014). The circulating Fe^3+^ and TF complex are endocytosed into cells by the membrane protein transferrin receptor 1 (TFR1). In the endosome, Fe^3+^ is reduced to Fe^2+^ by STEAP3, and Fe^2+^ is then released into unstable iron pools mediated by DMT1, or stored in ferritin, which is composed of FTL and FTH1 (Yang and Stockwell, 2008; Dixon et al., 2012). Excessive Fe^2+^ is exported through the membrane protein FPN and oxidized by ferroxidases, such as ceruloplasmin (Bogdan et al., 2016; Shang et al., 2020). In this process, iron accumulation (Shang et al., 2020) and administration of iron-bound, rather than iron-free TF, promote erastin-induced ferroptosis (Gao et al., 2015). On the contrary, using some iron chelators [e.g., DFP (Wu et al., 2020), DFO (Wu et al., 2018; Chen et al., 2020), and BPS (Codenotti et al., 2018)] may suppress ferroptosis and provide a potential therapeutic approach for diseases. In fact, there are some iron-chelating agents under clinical development for the treatment of cancers [for review see Brown et al. (2020)]. Moreover, inhibition of the IREB2 increases the expression of FTL and FTH1, thus decreasing sensitivity to ferroptosis (Gammella et al., 2015).

Although the importance of intracellular free iron in ferroptosis is confirmed, the regulatory mechanism of iron remains unknown. To date, the evidence has shown that the non-enzymatic free radical chain reaction involving Fenton Chemistry, in which Fe^2+^ is converted to Fe^3+^ with increased ROS (Winterbourn, 1995; He et al., 2020), and enzymatic processes (most notably the lipoxygenase pathway, LOXs), contributed to the formation of lipid peroxides in ferroptosis. Moreover, iron may promote ferroptosis through other iron-dependent enzymes, such as HIF-PHDs (Siddiq et al., 2009). Therefore, iron metabolism is one of the mechanisms of ferroptosis, and utilizing iron chelators to decrease iron may be useful for treating diseases.

## The Role and Mechanism of Ferroptosis in Acute CNS Injuries

**FIGURE 2:**
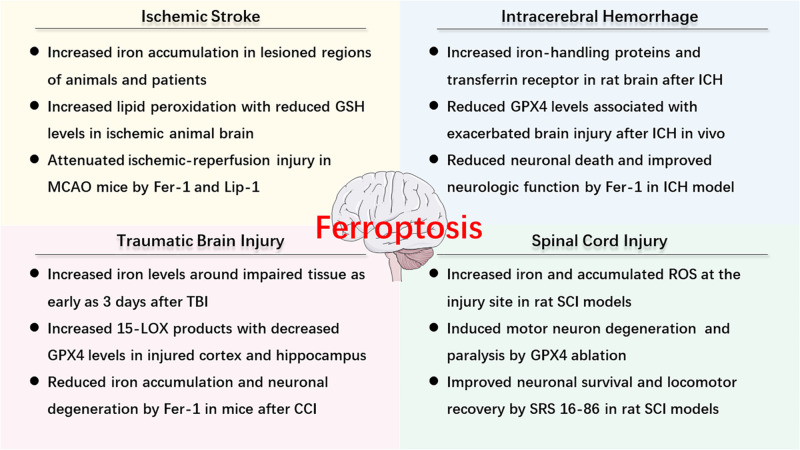
An overview of ferroptosis-associated mechanism and potential therapy in acute CNS injuries.

As described above, ferroptosis is an iron-dependent cell death that involves abnormal metabolism of lipids, GSH, and iron. The methods of measurement in evaluating ferroptosis in diverse diseases mainly depend on monitoring the levels of iron and lipid peroxidation, the activity of GPX4, as well as the ability of ferroptosis inhibitors (e.g., iron chelators, LOX inhibitors, and ferrostatins) to reduce cell death [for review see Xie et al. (2016)]. Observations of typical morphological features under a TEM also contribute to the distinguishing characteristics of ferroptosis compared to other cell deaths, both *in vitro* and *vivo* (Friedmann Angeli et al., 2014; Alim et al., 2019; Li et al., 2019d). Recently, numerous studies have confirmed the hypothesis of ferroptosis in the pathophysiology of acute CNS injuries, including stroke (Alim et al., 2019; Guan et al., 2019), TBI (Kenny et al., 2019; Xiao et al., 2019; Xie et al., 2019), and SCI (Dinc et al., 2013; Hu et al., 2017). More studies are included in the following text and Figure 2 is a brief summary of ferroptosis in acute CNS injuries.

### The Role and Mechanism of Ferroptosis in Ischemic Stroke

Ischemic stroke occurs when the blood flow to a certain portion of the brain is obstructed secondary to occlusion of cerebral arteries. The following deprivation of oxygen and energy triggers an ischemic cascade, such as OS and inflammation, resulting in neuronal excitotoxicity and cell death (Khoshnam et al., 2017; Li et al., 2019a; Zhang K. et al., 2019). Before ferroptosis was defined, iron accumulation [for review see Selim and Ratan (2004)] had been found in lesioned regions, such as the basal ganglia and the hippocampal area of the brain, and iron overload exaggerated neuronal damage during reperfusion (Dietrich and Bradley, 1988; Kondo et al., 1995; Lipscomb et al., 1998; Park et al., 2011). In MCAO animals, the iron intake was positively associated with the infarct volume (Castellanos et al., 2002; García-Yébenes et al., 2012). Consistently, one *in vitro* study demonstrated that holo-transferrin increased ROS production, and caused neuronal cell death induced by deprivation of oxygen and glucose (DeGregorio-Rocasolano et al., 2018). The experimental results also illustrated that administration of exogenous apotransferrin reduced brain damage and improved neurological outcomes with decreased lipid peroxidation, supporting the involvement of ferroptosis in ischemia (DeGregorio-Rocasolano et al., 2018). What’s more, the iron levels in the brain increased as humans age (Ward et al., 2014), which may exacerbate ischemic stroke. Recently, the tau-iron interaction has been proposed as an effective modulator of ferroptosis in ischemic stroke. The tau knockout mice were found to have increased protection from ferroptotic cell death following I/R injury, and the benefit of tau knockout was reinstated in older mice using iron-targeting interventions (Tuo et al., 2017). In this regard, iron chelation therapy reduced ischemic damage and improved outcome in mammals after ischemic stroke (Freret et al., 2006; Hanson et al., 2009). Consistently, Speer et al. (2013) found that the iron-dependent HIF-PHDs served as a target of metal chelators in ferroptosis, and the administration of iron chelators could inhibit HIF-PHDs, rather than suppress Fenton’s Reaction or ROS production, providing beneficial effects on subjects.

Additionally, other ferroptosis-associated mechanisms, such as LOXs-mediated pathology (Yang et al., 2016) and the GPX4-GSH-cysteine axis (Cho et al., 2007; Guan et al., 2019), were found to be involved in brain ischemia. MDA, a marker of oxidized lipids, was noticeably elevated, and this change correlated with increased activity of LOXs in an ischemic animal brain (Yigitkanli et al., 2013; Guan et al., 2019). Treatment with 12/15-LOX inhibitor (ML351) was shown to reduce infarct sizes and reperfusion damage in a mouse model (Rai et al., 2014). Moreover, Tuo et al. (2017) observed that brain damage was significantly attenuated by ferroptosis inhibitors, Lip-1, and Fer-1, in an MCAO model. As for the GPX4-GSH-cysteine axis, various groups provide direct evidence supporting that inhibition of system xc- induces ferroptosis and aggravates ischemia. Lan et al. (2020) found that acute cerebral ischemia-induced neuronal ferroptosis and treatment with Naotaifang increased the expression levels of xCT, GPX4 and GSH, and the number of Nissl bodies in MCAO rats. These data suggested that Naotaifang may rescue ischemic stroke by inhibiting ferroptosis through the xCT/GPX4 pathways (Lan et al., 2020). Huang et al. (2019) also observed that inhibition of system xc- with erastin aggravated ferroptosis and augmenter of liver regeneration protected the kidney from ischemia-reperfusion injury in ferroptosis through GSH/GPX system. Guan et al. (2019) and Cho et al. (2007) identified reduced levels of GSH and decreased activities of GPX4 in ischemia. However, many researchers hold opposing views and state that activated system xc- can exacerbate ischemic cerebral injury due to increased glutamate. In their studies, xCT was expressed in significant concentrations in astrocytes in the mouse brain (Jackman et al., 2012; Thorn et al., 2015; Ottestad-Hansen et al., 2018). Increased activity of system xc-(Soria et al., 2014) promoted the release of glutamate which may contribute to excitotoxicity in pathological situations (e.g., oxygen and glucose deprivation), leading to neuronal death (Thorn et al., 2015; Ottestad-Hansen et al., 2018). Hsieh et al. (2017) used both *in vitro* and *in vivo* models to reveal that HIF-1α triggered long-lasting glutamate excitotoxicity via activation of system xc- dependent glutamate outflow. HIF-1α conditional knockout mouse had reduced extracellular glutamate in cerebral ischemia-reperfusion, suggesting that system xc- was a promising therapeutic target (Hsieh et al., 2017). Therefore, inhibition of system xc- can induce ferroptosis to promote neuronal death due to GSH depletion and activation of system xc- can also increase neuronal death because of glutamate-associated excitotoxicity. Whether induction of system xc- activity is beneficial or detrimental might depend on the pathway of induction and whether inhibition of system xc-or induction of xCT is the more promising neuroprotective strategy remains to be explored.

Other studies have confirmed the benefits of the necroptosis inhibitor, necrostatin-1, indicating that alternative forms of regulated cell death were involved in ischemic brain injury (Degterev et al., 2005). However, necrostatin-1 was later found to protect against ferroptosis through an unknown target (Friedmann Angeli et al., 2014). These results suggest that there is the direct involvement of ferroptosis in the pathogenesis of ischemic stroke. What about ferroptosis in hemorrhagic stroke?

### The Role and Mechanism of Ferroptosis in ICH

After ICH, there is a consequent physical disruption of the neurovascular architecture due to the mass effects and elevated pressure surrounding hemorrhagic sites, inducing primary brain injury. Subsequently, iron accumulates as a result of the degradation of Hb and its metabolite, hemin, which contributes to SBI (Hu et al., 2016). Previous data have identified multiple forms of cell death after ICH, including necrosis, apoptosis, and autophagy (Qureshi et al., 2001, 2003; Wang et al., 2015; Li et al., 2018). To date, multiple laboratories have provided converging lines of evidence that support the role of ferroptosis in ICH with the presence of observed molecular markers and morphological features (Li et al., 2017; Zille et al., 2017; Zhang et al., 2018; Alim et al., 2019), and the underlying mechanisms of ferroptosis in ICH are analogous to those of ischemic stroke. It was identified that iron overload stimulated neuronal ferroptosis, which aggravated brain damage (Wu et al., 2003, 2011). Besides, as one of the major upstream regulators of ferroptosis, GPX4 inactivation also contributes to ICH (Forcina and Dixon, 2019). It was found that levels of GPX4 were reduced and brain injury was exacerbated in a rat model of ICH induced by autologous blood injection, whereas overexpression of GPX4 was able to alleviate SBI and improve neurological outcomes (Zhang et al., 2018). Li et al. (2017) also indicated that administration of Fer-1 reduced Hb-induced cell death and iron deposition, prevented impairment of GPX4 activity i*n vitro*, and improved neurologic function in collagenase-induced ICH models.

Many recent studies have suggested potential approaches to reduce brain damage. Zille et al. (2017) revealed that several ferroptosis inhibitors, including Fer-1, DFO, Trolox (a lipid peroxidation inhibitor), and NAC (a cell-permeable cysteine analog), were able to alleviate Hb- and hemin-induced cell death *in vitro*. Alim et al. (2019) also supported the role of Fer-1 by showing that inhibition of ferroptosis by Fer-1 exerted a long-term cerebroprotective effect in *in vitro* and *in vivo* ICH models. Dharmalingam et al. (2020) synthesized a multifunctional nanoparticle that protected cells from both senescence and ferroptosis, leading to a reduction of hemin/iron-induced toxicity in experimental ICH. In addition, Karuppagounder et al. (2018) found that ALOX5 inhibition could protect against ICH- or hemin-induced ferroptosis *in vivo* following ICH.

Furthermore, the role of ferroptosis in ICH is supported by altered levels of other ferroptosis-related molecules. For example, phospho-ERK1/2, regarded as a molecular feature of ferroptosis (Yagoda et al., 2007), was significantly increased in mice with ICH, but the MEK inhibitor, U0126, inhibited this type of cell death (Zille et al., 2017). In addition, the expression levels of PTGS2 were also significantly increased in *in vitro* and *in vivo* ICH models (Li et al., 2017; Alim et al., 2019). Moreover, PTGS2 has been revealed as part of the downstream signaling pathway of ferroptosis in cancer cells (Yang et al., 2014). Notably, a gene product of PTGS2, known as COX-2, was substantially increased in neurons after ICH, and treatment with Fer-1 could reduce its expression and ICH-induced SBI, implying that COX-2 might be used as a biomarker of ferroptosis (Zhao et al., 2007; Li et al., 2017; Alim et al., 2019). Thus far, there is little evidence that shows the relationship between ferroptosis and subarachnoid hemorrhage. More studies are warranted to investigate this promising topic.

### The Role and Mechanism of Ferroptosis in TBI

The previous literature has shown that TBI shares many mechanisms (e.g., OS, inflammation, mitochondrial dysfunction, and neuronal cell death) with stroke (Blennow et al., 2016). In addition to these mechanisms, multiple studies have demonstrated that ferroptosis may contribute to the neuronal cell death and functional outcome in TBI (Ayton et al., 2014; Stockwell et al., 2017). The altered levels of various ferroptosis biomarkers provide evidence of ferroptosis in TBI. Studies have detected elevated iron concentrations around the impaired tissue as early as 3 days after injury, occurring in adult and aged mice models of controlled CCI (Portbury et al., 2016, 2017; Xie et al., 2019). The evidence also suggested that increased iron accumulation was negatively associated with cognitive outcomes in chronic TBI patients (Lu et al., 2015), while iron chelators exhibited neuroprotective effects by diminishing iron-mediated brain damage (Zhang et al., 2013; Khalaf et al., 2018). According to recent studies, ferroptosis was identified in TBI through the detection of ferroptosis-associated molecules, such as 15-LOX and GPX4. For instance, there were enhanced levels of 15-HpETE-PE and 15-LOX2 in the injured cortex and ipsilateral hippocampus, and decreased levels of GPX4 in a pediatric rat CCI model, suggesting that ferroptosis might occur within the first hour after TBI (Wenzel et al., 2017). Researchers also showed a preponderance of 15-LOX products in CCI-injured adult mice, and increased ACSL4 and 15-LOX2 expression in TBI, when compared with naive groups (Kenny et al., 2019). Moreover, numerous experimental and clinical studies observed the increased levels of MDA and 4-HNE in either the injured brain or serum following TBI (Hall et al., 2004; Readnower et al., 2010; Lorente et al., 2015; Xiao et al., 2019; Xie et al., 2019). As for the morphological features, Xie et al. (2019) first verifiably identified the ferroptotic features around brain injury lesions of TBI models at 3 days after CCI. As ferroptosis was shown to participate in TBI by the above work, inhibiting ferroptosis may be useful in treating TBI. For example, treatment with Fer-1 significantly diminished iron accumulation, reduced neuronal cell death, and attenuated neuronal degeneration (Kenny et al., 2019; Xie et al., 2019). More details regarding therapy can be found in the next section.

### The Role and Mechanism of Ferroptosis in SCI

In traumatic SCI, the primary injury causes immediate cellular damage and initiates a continuous secondary injury cascade to induce ischemia, inflammation, and cell death [for review see Ahuja et al. (2017)]. Notably, following the rupture of the blood-spinal cord barrier and blood vessels, hemorrhage occurs in the acute phase of SCI and may last for days (Tran et al., 2018). Like other acute CNS diseases, ferroptosis occurs in SCI, and is accompanied by increased iron and accumulated ROS at the site of injury (Liu et al., 2011; Visavadiya et al., 2016; Hao et al., 2017), as well as excessive lipid peroxidation (Dinc et al., 2013; Hu et al., 2017). This phenomenon is more apparent during the first several hours (Liu et al., 2004). In adult mouse models, Chen et al. (2015) observed that conditional ablation of Gpx4 in neurons could induce motor neuron degeneration and cause rapid paralysis, but this result was delayed by supplementation with vitamin E, suggesting that ferroptosis accelerated SCI. Therefore, anti-ferroptotic seems to have potential in SCI, though there are few studies. Feng et al. (2019) established a rat model of DFO and confirmed the positive role of DFO in treating SCI. In the DFO group, there were lower iron concentrations, markedly increased GPX4 expression, and increased neuronal survival (Feng et al., 2019). In the study conducted by Zhang Y. et al. (2019) treatment with the third-generation ferrostatin, SRS 16-86, increased neuronal survival and promoted locomotor recovery in the SCI model, providing potential therapeutic strategies for SCI. Indeed, it is well-known that the excitotoxicity caused by glutamate accumulation preceded neuronal death and reuptake failure of astrocytes, and also induced ferroptotic cell death to stimulate secondary injury after SCI (Dixon et al., 2014; Ahuja et al., 2017). The relationship between ferroptosis or excitotoxicity in SCI requires further studies.

## Potential and Emerging Therapy Targeting Ferroptosis in Acute CNS Injuries

As ferroptosis may be a significant pathogenic pathway in acute CNS injuries, its therapeutic potential should be taken into consideration. Ferroptosis inhibitors (including iron chelators, ferrostatins, liproxstatins, LOX inhibitors, and antioxidants) may prevent iron accumulation or lipid peroxidation, thus offering therapeutic options for treating acute CNS injuries (Table 1).

**TABLE 1:** Ferroptosis-associated drugs in treating the acute CNS injuries.

**Disease**	**Drug**	**Type**	**Administration Route**	**Function and Mechanism**
Ischemic stroke	Liproxstatin-1 (Lip-1)	Lipid peroxides inhibitor	Intranasal	Attenuated motor function deficits, cognitive impairment; improved neuroscores; reduced infarct volumes in middle cerebral artery occlusion (MACO) mice.
	Ferrostatin-1 (Fer-1)	Lipid peroxides inhibitor	Intranasal	Attenuated neurological deficits and infarct volumes in MACO mice.
	ML351	Inhibitor of 15-lipoxygenase-1	Intravenous	Reduced neurological impairment and infarct volume in MACO mice.
	Amyloid precursor protein ectodomain	Protein stabilizing ferroportin to export iron	Intravenous	Improved neuroscores and infarct volume; prevented iron accumulation in the lesioned hemisphere in MACO mice.
	Ceruloplasmin	Copper regulating iron-mediated transport	Intraperitoneal	Suppressed ischemia-induced hippocampal iron elevation in the lesioned hemisphere in MACO mice.
	Carvacrol	Monoterpenic phenol	Intraperitoneal	Reduced neuronal cell death, increased GPx4 expression in gerbils I/R hippocampal neurons (*in vitro*); decreased the level of lipid peroxide and MDA, TFR, increased the Fpn1 expression; alleviated neuronal degeneration and memory deficits in I/R gerbils.
	Deferoxamine	Iron chelator	Intraperitoneal	Suppressed the level of MDA.
	Edaravone	Free radical scavenger; **A clinically approved drug for treating acute ischemic stroke**	Not applicable	Suppressed the accumulation of lipid peroxidation and ROS production; inhibited ferroptosis induced by cystine deprivation, erastin and RSL3 by scavenging radical species in non-neuronal cells (*in vitro*).
	Tat-linked SelP Peptide	BBB-permeable peptide containing selenocysteine	Intraperitoneal	Reduced infarct volume in rodent MCAO model.
ICH	Ferrostatin-1	Lipid peroxides inhibitor	Intracerebroventricular or intraperitoneal	Prevented lipid ROS, MDA and GPx activity deficit (*in vitro*); inhibited Hb/ferrous-induced and hemin/hemoglobin-induced neuronal death (*in vitro*); reduced iron deposition and lipid ROS; diminished injury volume; rescued degenerating neurons, and corrected neurologic deficit in collagenase-induced ICH model; suppressed the level of GPX4; alleviated neuronal dysfunction; moderated brain atrophy and exerted long-term neuroprotective effects in autologous blood infusion model of ICH.
	Liproxstatin-1	Lipid peroxides inhibitor	Intraperitoneal	Inhibited Hb-induced cell death; decreased neurologic deficits and lesion volume; rescued neuronal cells in collagenase-induced ICH model.
	Zileuton/BW B70/BW A4C	Arachidonate 5-lipoxygenase (ALOX5) inhibitors	Not applicable	Inhibited Hb/hemin-induced cell death (*in vitro*).
	Compound 968	Glutaminase inhibitor	Intraperitoneal	Decreased degenerating neurons.
	Deferoxamine	Iron chelator		Inhibited hemin/hemoglobin-induced neuronal death.
	N-acetylcysteine (NAC)	Glutathione prodrug; Thiol-containing redox modulatory compound	Intraperitoneal	Inhibited hemin/hemoglobin-induced neuronal death (*in vitro*); increased glutathione, deceased nuclear ALOX5-derived reactice lipid species, reduced neuronal death, improved functional recovery in collagenase-induced mouse model of ICH.
	Trolox	Water soluble lipid peroxidation inhibitor	Not applicable	Inhibited hemin/hemoglobin-induced neuronal death (*in vitro*).
	U0126	Extracellular-signaling kinase 1/2 (ERK1/2) inhibitor	Not applicable	Inhibited hemin/hemoglobin-induced neuronal death (*in vitro*).
	(-)-Epicatechin	Brain-permeable flavanol	Orally	Diminished heme oxygenase-1 expression and brain iron deposition via an Nrf2-independent pathway, reduced lesion volume and ameliorated neurologic deficits in collagenase/autologous blood/thrombi-induced ICH model.
	Loxothiazolidine-4-carboxylate (OTC)	Cysteine prodrug	Not applicable	Inhibited hemin-induced neuronal death (*in vitro*).
	Glutathione ethyl ester	Membrane permeable form of glutathione	Not applicable	Inhibited hemin-induced neuronal death (*in vitro*).
	Tat SelPep	BBB-permeable peptide containing selenocysteine	Intraperitoneal	Increased GPX4 expression; prevented hemin-induced ferroptosis and preserved cell bodies and neurites of neurons (*in vitro*); unregulated transcriptional expression of GPX4; inhibited cell death and improved function in collagenase-induced ICH model.
	Selenium	Essential micronutrient	Intracerebroventricular	Diminished cell death and improved functional recovery in a mouse model of ICH.
TBI	Ferrostatin-1	Lipid peroxides inhibitor	Intracerebroventricular	Reduced neuronal death in mechanical stretch-elicited TBI model (*in vitro*); reduced iron accumulation, neuron degeneration and lesion volume; ameliorated cognitive and motor function deficits in the adult controlled cortical impact injury (CCI) mouse model.
	Triacsin C	Acyl-CoA synthetase long-chain family member 4 (ACSL4) inhibitor	Not applicable	Reduced neuronal cell death in mechanical stretch-elicited TBI model (*in vitro*).
	Liproxstatin-1	Lipid peroxides inhibitor	Not applicable	Reduced neuronal cell death in mechanical stretch-elicited TBI model (*in vitro*).
	Baicalein	12/15-lipoxygenase inhibitor	Intraperitoneal	Reduced neuronal cell death in mechanical stretch-elicited TBI model (*in vitro*); attenuated phosphatidylethanolamine oxidation and improved function in CCI mouse model.
	miR-212-5p agomir	MicroRNAs agomir	Intracerebroventricular	Improved memory and learning in CCI mice.
SCI	Deferoxamine	Iron chelator	Intraperitoneal	Increased xCT, GSH, and GPX4 levels; protected neurons and promoted long-term functional recovery in rat contusion SCI model.
	SRS 16-86	Small molecule ferroptosis specific inhibitor	Intraperitoneal	Upregulated GPX4, GSH and xCT levels; down-regulated the expression of 4HNE; increased neuronal survival and promoted functional recovery in rat contusion SCI model.

### Targeting Ferroptosis Therapy in Ischemic Stroke

The mainstay of treatment for acute ischemic stroke is rapid recanalization by mechanical thrombectomy or recombinant tissue plasminogen activator, the only approved thrombolytic agent. It is important to salvage the penumbra, which surrounds the region of the infarct and promotes functional recovery. However, the overall efficacy is limited due to the narrow window of opportunity (Sandercock et al., 2012), but even after timely recanalization, infarct volume often continues to increase in I/R injury (Nour et al., 2013). As previous methods have failed in clinical use, such as blocking excitotoxicity, the role of ferroptosis has been highlighted (Khoshnam et al., 2017; Tuo et al., 2017), and new therapeutic approaches targeting ferroptosis or combined therapies are highly desirable.

As mentioned above, Lip-1 and Fer-1 are both compounds with specific anti-ferroptotic activity. Intranasal administration of Lip-1 and Fer-1, either immediately or 6 h after reperfusion, significantly reduced neuronal damage and functional deficits in MCAO mice, indicating the possible translational value of special exogenous ferroptosis inhibitors (Tuo et al., 2017).

In addition, CoQ10 is an endogenous lipid-soluble antioxidant with established efficacy in suppressing the initiation and amplification of lipid peroxidation (Morris et al., 2013; Viswanathan et al., 2017), presenting a promising candidate for ferroptosis inhibition. Intriguingly, *in vivo* studies have reported that oral CoQ10 administration markedly improved neurological outcomes in both rat MCAO models and acute ischemic stroke patients (Ramezani et al., 2018; Nasoohi et al., 2019). This neuroprotective benefit of CoQ10 was associated with its anti-apoptotic effect, as the levels of peroxidation products were not altered (Nasoohi et al., 2019). The authors attributed this to a relatively higher dose and multiple potential mechanisms of CoQ10. Of note, it is worth considering whether inhibition of ferroptosis is involved.

Recently, Guan et al. (2019) found that carvacrol, a plant-derived monoterpenic phenol, inhibited hippocampal neuronal damage and reduced functional deficits in gerbils following I/R injury. Furthermore, treatment with carvacrol (100 mg/kg, intraperitoneally) for two consecutive weeks after reperfusion was associated with decreased ROS, reduced iron overload, and increased levels of GPX4, suggesting a possible neuroprotective role of carvacrol via ferroptosis inhibition (Guan et al., 2019). Carvacrol is thought to easily cross the BBB because of the small molecular weight and the lipophilic profile (Savelev et al., 2004). A previous study has proven the benefits of carvacrol when administered intraperitoneally at 2 h after reperfusion. When administered intracerebroventricularly, the treatment window was prolonged to 6 h (Yu et al., 2012). When administered safely, carvacrol may be regarded as a potential therapeutic agent.

Edaravone is an effective radical scavenger that inhibits lipid oxidation by scavenging chain-initiating water-soluble radicals and chain-carrying lipid peroxyl radicals due to its amphiphilicity (Watanabe et al., 2018). There are several papers describing its alleviatory effects on neurological symptoms in ischemia models, and its effective treatment window of at least 3 h after embolism (Nishi et al., 1989; Kawai et al., 1997; Lapchak and Zivin, 2009). In the clinical setting, the appropriate dosage and course of edaravone use for patients suffering from acute ischemic stroke include intravenous administration of 60 mg daily for up to 14 days (Feng et al., 2011). Homma et al. (2019) recently indicated that edaravone participates in rescuing ferroptotic cell death induced by cystine deprivation, erastin, and RSL3 (Homma et al., 2019). In addition, edaravone was confirmed to suppress the accumulation of Fe^2+^ and lipid peroxidation *in vitro*, which are known as the metabolic characteristics of ferroptosis.

### Targeting Ferroptosis Therapy in ICH

Currently, there are no proven medical or surgical treatments that substantially improve the neurological outcomes in patients with ICH because of multiple underlying mechanisms, including inflammation, excitotoxicity, and OS (Keep et al., 2012). As emerging studies suggest that ferroptosis is involved in SBI after ICH, and contributes to 80% of whole-cell death *in vitro* (Li et al., 2017; Zille et al., 2017), ferroptosis-based treatments could be highly considered.

Li et al. demonstrated the neuroprotective effects of Fer-1 by striatum injection immediately after and by cerebral ventricular injection 2 h after collagenase-induced ICH [for review see Li et al. (2017)]. Intraperitoneal administration of Fer-1 (a 3-h delay and then once daily) in the autologous blood infusion model of ICH was also shown to improve long-term neurological function (Alim et al., 2019). Moreover, when combined with other inhibitors of either apoptosis or necrosis, Fer-1 was found to be more effective at reducing Hb-induced cell death, which should be further investigated in *in vivo* models (Li et al., 2017).

Various evidence has shown that iron chelators reduce Hb- and iron-induced neurotoxicity attenuates brain edema, and improve functional neurologic outcomes after ICH (Nakamura et al., 2004; Wu et al., 2012; Hatakeyama et al., 2013). A meta-analysis of 20 studies involving animal models of ICH revealed that DFO was neuroprotective, particularly when administered 2–4 h after ICH induction (Cui et al., 2015), whereas there remains a lack of conclusive clinical evidence regarding iron chelators (Zeng et al., 2018).

N-acetylcysteine (Gilbert et al., 1991; Leslie et al., 1992; Varga et al., 1997) is an FDA-approved cysteine prodrug capable of regulating the activity of system xc- and the biosynthesis of GSH [for review see Berk et al. (2013)]. Interestingly, a recent study reported that systemic administration of NAC post-injury reduced neuronal death and improved behavior following ICH in mice (Karuppagounder et al., 2018). They further pointed out that NAC inhibited hemin- and ICH-induced ferroptosis by neutralizing nuclear ALOX5-derived toxic lipid species (Karuppagounder et al., 2018). This process relied on increased GSH and enhanced activity of GSH-dependent antioxidant enzymes. Considering the poor absorption of direct GSH administration and the insufficient capacity of GSH to cross the BBB (Witschi et al., 1992), NAC may be treated as an adjuvant therapy candidate capable of penetrating the BBB to enter the brain (Farr et al., 2003).

In addition, EC, a brain-permeable flavanol, was shown to reduce early brain injury and improve neurologic deficits in multiple experimental ICH models when administered orally at 3 h post-treatment and subsequent daily administration (Chang et al., 2014). The neuroprotective effects of EC were partially associated with decreased iron deposition and modulation of ferroptosis-related gene expression, indicating the possible ability of EC to inhibit ICH-induced ferroptotic cell death (Chang et al., 2014).

Se is indispensable for the ferroptosis-resistant function of GPX4 (Ingold et al., 2018). It was recently uncovered that Se could amplify an adaptive transcriptional program response to neuronal ferroptosis (Alim et al., 2019), making it a potential therapeutic strategy. Further studies discovered that injection of Se directly into the mouse cerebral ventricle following ICH was associated with elevated GPX4 levels, diminished ferroptotic death, as well as improved functional recovery. Moreover, the researchers developed a peptide (Tat SelPep), which contained a Tat transduction domain combined with selenoprotein P. Intraperitoneal injection of Tat SelPep showed similar effects compared to Se, but with reduced toxicity and a wider treatment window, with benefits shown even at 6 h post-injury (Alim et al., 2019).

### Targeting Ferroptosis Therapy in TBI

When contemplating feasible treatments for TBI, researchers focus on secondary events, which cause delayed damage, to provide applicable therapeutic windows for interventions [for review see Lozano et al. (2015)]. Given that there are currently no effective treatments approved by clinical trials for TBI patients (Pearn et al., 2017), there exists a pressing need for developing more innovative methods, such as targeting ferroptotic cell death in a highly regulated manner. The ferroptosis signaling molecules can be prevented as a result of reducing PE oxidation by inhibiting the ability of 15LOX/PEBP1 complexes to produce 15-HpETE-PE, administrating 15LOX inhibitors, or augmenting the GPX4/GSH system to remove oxidized PE products (Wenzel et al., 2017; Kenny et al., 2019).

Baicalein is a polyphenolic antioxidant 12/15-LOX inhibitor and is well-known to exert neuroprotective effects in cerebral ischemia [for review see Liang et al. (2017)]. Recently, Kenny et al. (2019) demonstrated that baicalein decreased the accumulation of pro-ferroptotic PE oxidation, but not pro-apoptotic cardiolipin oxidation after CCI, indicating that the 15-LOX inhibitory effects of baicalein may have an anti-ferroptotic role in TBI. Several studies have also revealed a reduction of functional and histological damage with the immediate administration of baicalein post-injury (Chen et al., 2008; Kenny et al., 2019). With low levels of toxicity and the ability to cross the BBB (Tsai et al., 2002), baicalein offers great promise in clinical settings if the effect of delayed drug delivery is evaluated.

As mentioned above, NAC is a precursor for GSH, and it has been shown to confer antioxidant and neuroprotective effects after pre-clinical TBI (Eakin et al., 2014; Senol et al., 2014). As for adult patients, a double-blinded and placebo-controlled study indicated that supplementation of oral NAC had significant short-term benefits on neurological symptoms and sequelae resolution after blast-induced mild TBI (Hoffer et al., 2013). Due to the low bioavailability of NAC, the compound, NACA, was developed with increased membrane permeability, and its neuroprotection was associated with the activation of the Nrf2-antioxidant response elements signaling pathway in a mouse model of TBI (Zhou et al., 2018). It is well-established that Nrf-2 regulates xCT and GPX4, whose inhibition initiates ferroptosis and promotes target genes that mediate the antioxidant and iron metabolic status of cells (Zhou et al., 2018), suggesting another anti-ferroptotic mechanism of NACA.

In a mouse CCI model of TBI, Fer-1 treatment had been injected directly into the cerebral ventricle 0.5 h after injury, causing a reduction in neuronal death and other associated functional defects (Xie et al., 2019). However, more research should be implemented to uncover feasible drug-delivery methods.

Moreover, a recent study demonstrated the role of miR-212-5p in suppressing ferroptosis after TBI, partially by targeting PTGS2 (Xiao et al., 2019). Further results showed that intracerebroventricular injection of miR-212-5p agomir improved spatial memory and learning in CCI mice, suggesting that miR-212-5p may serve as a potential ferroptosis inhibitor to be used in treating TBI. As previously discussed, the oxidation of AA/AdA-PE is a critical step in ferroptosis execution. Therefore, inhibition of ACSL (such as triacsin C and thiazolidinedione) and formation of AA/AdA-esterified PE may also protect against ferroptosis after TBI (Doll et al., 2017; Kagan et al., 2017; Kenny et al., 2019).

### Targeting Ferroptosis Therapy in SCI

There are no neuroprotective or neurodegenerative strategies currently approved for acute traumatic SCI, but several are currently undergoing clinical trials (Badhiwala et al., 2018). The concept of “time is spine” is commonly applied in the management of patients with SCI. Since ferroptosis is likely involved in the acutes phases of SCI, therapies targeting ferroptosis are promising (Badhiwala et al., 2018; Zhang Y. et al., 2019).

Targeting iron is one of the treatment strategies. The iron chelator, DFO, reportedly reduced iron accumulation and lipid peroxidation, while modulating the inflammatory response in SCI (Paterniti et al., 2010; Liu et al., 2011; Dinc et al., 2013; Hao et al., 2017). Experimental evidence indicated that DFO improved motor function recovery when injected intraperitoneally post-SCI (Paterniti et al., 2010; Liu et al., 2011; Hao et al., 2017). Moreover, DFO showed neuroprotective effects comparable with methylprednisolone, an effective antioxidant agent that is contentious for the treatment of SCI because of harmful side effects (Dinc et al., 2013; Silva et al., 2014). However, oral treatment of deferasirox, another FDA-approved iron chelator, failed to remove iron from the injured spinal cord, but markedly depleted the systemic iron (Sauerbeck et al., 2013). Considering the detrimental side effects (e.g., anemia) and the absence of potent neuroprotection, systemic administration may not be the ideal approach of iron chelators (Grossman et al., 2012; Sauerbeck et al., 2013).

Besides, previous studies have demonstrated that NAC administration suppressed OS, attenuated neuroinflammation, and improved neuronal survival and neurological recovery following SCI in rodent models (Karalija et al., 2012, 2014; Guo et al., 2015). When administered immediately after SCI, NACA, an amide derivative of NAC, improved mitochondrial function, antioxidant GSH levels, and functional recovery in SCI mice (Patel et al., 2014). These two GSH precursors facilitate the biosynthesis of intracellular GSH. In addition, the GSH antioxidant system plays a pivotal role in the regulation of ferroptosis (Lv et al., 2019).

Recently, Li et al. (2019b) observed that CoQ10, a promising ferroptosis inhibitor previously mentioned, exerted protective effects by decreasing OS partly through activation of the Nrf-2 signaling pathway after SCI. Moreover, Nrf-2 is regarded as a significant mitigator of lipid peroxidation and ferroptosis [for review see Dodson et al. (2019)]. Furthermore, CoQ10 was shown to protect BMSCs from OS, and improved the therapeutic efficacy in combination with BMSC transplantation, suggesting a promising therapy for SCI (Li et al., 2019c).

Besides, post-injury intraperitoneal injection of SRS 16-86 proved to be more potent and stable than Fer-1. It also attenuated the ferroptotic mitochondrial morphology in damaged areas, and improved neurological deficits in SCI model, suggesting the role of ferroptosis-specific inhibitors in the treatment of SCI (Zhang Y. et al., 2019).

## Discussion and Perspective: Will Ferroptosis Be the Future Direction?

In this article, we primarily focus on the roles and therapeutic potential of ferroptosis in various acute CNS injury processes, including stroke, TBI, and SCI. Pharmacological effects of multiple inducers and inhibitors of ferroptosis lie at the intersection of lipid, amino acid, and iron metabolism. Although some progress has been made in ferroptosis, there are still controversial questions that have not been fully studied. First, the relationship between ferroptosis and other forms of cell death remains unknown. For example, p53 is an important regulator, both in apoptosis and ferroptosis, while autophagy plays a role in the process of ferroptosis via ferritinophagy. As ferroptosis is involved in acute CNS injuries complicated by necrosis, apoptosis, and autophagy, a head-to-head comparison of individual inhibitors or various combinations of inhibitors is required in further studies. Second, the special molecular markers (e.g., caspase activation for apoptosis or the autophagosome marker, LC3-II, for autophagy) for identifying ferroptosis are still lacking. While the increased mRNA levels of PTGS2 were found in cells undergoing ferroptosis, it did not affect ferroptosis progress (Yang et al., 2014). The specificity of PTGS2 expression or its gene product, COX-2, for ferroptosis needs to be explored in the context of different pathophysiologic processes. Actually, there is copious evidence for the role of COX-2 in several acute neurological disorders (e.g., ischemic and hemorrhagic strokes) (Gong et al., 2001; Tomimoto et al., 2002). The research of additional ferroptosis markers is of great importance for *in vivo* studies in the future. Moreover, the exact role of iron and the final molecular executor in ferroptosis remains unclear. Considering the complexity of the CNS, the biochemical regulation, as well as the sensitivity of ferroptosis in different cell types (neurons, astrocytes, microglia, or oligodendrocytes), also requires explication.

Potential treatment options targeting ferroptosis (e.g., iron chelators, ferrostatins, NAC, and CoQ10) have shown neuroprotective effects in acute CNS injuries. However, these benefits are largely based on animal models and have not yet translated into clinical application. Furthermore, studies are necessary to clarify the appropriate therapeutic window, clinically feasible routes of administration, and BBB penetration ability of anti-ferroptotic agents. Among the above-mentioned agents, edaravone is the only approved drug with proven clinical efficacy and safety, while others should be explored in further clinical studies. More in-depth and comprehensive research on ferroptosis should be conducted to develop therapeutic methods and eventually alleviate the burden of acute CNS injuries in the future.

## Author Contributions

All the authors participated in analyzing and discussing the literature, commenting on, and read and approved the final manuscript. AS and YC supervised the research, led the discussion, and wrote and revised the manuscript.

## Conflict of Interest

The authors declare that the research was conducted in the absence of any commercial or financial relationships that could be construed as a potential conflict of interest.

## Abbreviations

4-HNE: 4-hydroxy-2-nonenal
AA: arachidonic acid
ACSL4: acyl-CoA synthetase long-chain family member 4
AdA: adrenic acid
ALOX5: arachidonate 5-lipoxygenase
BBB: blood–brain barrier
BMSC: bone marrow mesenchymal stem cells
BPS: bathophenanthrolinedisulfonic acid
BSO: buthionine sulfoximine
CCI: cortical impact injury
CNS: central nervous system
CoQ10: coenzyme Q10
COX-2: cyclooxygenase-2
DFO: deferoxamine
DFP: deferiprone
DMT1: divalent metal transporter 1
EC: (-)-epicatechin
Fe^2+^: ferrous iron
Fe^3+^: ferric iron
Fer-1: ferrostatin-1
FPN: ferroportin
FSP1: ferroptosis suppressor protein 1
FTH1: ferritin heavy chain 1
FTL: ferritin light chain
GPX4: glutathione peroxidases 4
GSH: glutathione
Hb: hemoglobin
HIF-1 α: hypoxia-inducible factor 1 α
HIF-PHD: hypoxia-inducible factor prolyl hydroxylase
I/R: ischemia/reperfusion
ICH: intracerebral hemorrhage
IREB2: iron metabolism essential factor iron response element binding protein 2
Lip-1: liproxstatin-1
LOX: lipoxygenases
LPCAT3: lysophosphatidylcholine acyltransferase 3
MCAO: middle cerebral artery occlusion
MDA: malondialdehyde
NAC: N-acetylcysteine
NACA: N-acetylcysteine amide
Nrf2: nuclear factor erythroid 2-related factor
OS: oxidative stress
PEBP1: phosphatidylethanolamine-binding protein 1
PTGS2: prostaglandin-endoperoxide synthase 2
PUFA: polyunsaturated fatty acid
ROS: reactive oxygen species
RSL3: RAS synthetic lethal 3
SBI: secondary brain injury
SCI: spinal cord injury
Se: selenium
STEAP3: six-transmembrane epithelial antigen of the prostate 3
TBI: traumatic brain injury
TEM: transmission electron microscopy
TF: transferrin
TFR: transferrin receptor.
